# HOXC13-driven TIMM13 overexpression promotes osteosarcoma cell growth

**DOI:** 10.1038/s41419-023-05910-0

**Published:** 2023-07-05

**Authors:** Qicai Han, Penghui Yan, Ruipeng Song, Feifei Liu, Qing Tian

**Affiliations:** Department of Orthopaedics, The First Affiliated Hospital of Zhengzhou University, Zhengzhou, China

**Keywords:** Bone cancer, Targeted therapies

## Abstract

TIMM13 (translocase of inner mitochondrial membrane 13) located at the mitochondrial intermembrane space is vital for the integrity and function of mitochondria. We found that the mitochondrial protein TIMM13 is upregulated in human OS tissues and cells. In patient-derived primary OS cells and established cell lines, TIMM13 shRNA or knockout provoked mitochondrial dysfunction, causing mitochondrial depolarization, reactive oxygen species production, and oxidative injury, as well as lipid peroxidation, DNA damage, and ATP depletion. Moreover, TIMM13 depletion provoked OS cell apoptosis and inhibited cell proliferation and migration. Conversely, ectopic TIMM13 overexpression increased ATP contents, enhancing OS cell proliferation and migration. Moreover, we discovered that Akt-mTOR activation was inhibited with TIMM13 depletion in primary OS cells. Further studies revealed that HOXC13 (Homeobox C13)-dependent TIMM13 transcription was significantly increased in OS tissues and cells. Whereas TIMM13 transcription and expression were decreased following HOXC13 silencing in primary OS cells. In vivo, TIMM13 KO potently inhibited OS xenograft growth in the proximal tibia of nude mice. TIMM13 KO also induced Akt-mTOR inactivation, ATP depletion, oxidative injury, and apoptosis in the in situ OS tumors. Together, upregulation of the mitochondrial protein TIMM13 is important for OS cell growth, representing a novel and promising therapeutic target.

## Introduction

Osteosarcoma (OS) is the most common primary malignant bone tumor. Mainly detected among children and young adults, OS has an average patient age of 20 [1–4]. It accounts for 2.4% of all cancers in pediatric patients and is broadly classified into three different histological subtypes, including intramedullary OS, superficial OS, and extraosseous OS [1–4]. The high-grade intramedullary OS is the most common type of OS, accounting for approximately 80% of OS [1–4]. The most common bone sites for OS are the distal femur and the proximal tibia [1–4].

Tumor resection and chemotherapy are the current clinical treatments for OS patients [1, 5–7]. Immunotherapy could be utilized to treat high-grade undifferentiated pleomorphic OS [8]. Recurrent OS could possibly be controlled with tumor resection combined with radiotherapy, chemotherapy, and targeted therapies [1, 5–7]. Yet, for the advanced, recurrent, and metastatic OS, the 5-year overall survival (close to 20–30%) is far from satisfactory [9–13]. Therefore, novel molecular targets and therapeutic strategies are urgently needed to possibly overcome the drawbacks of conventional treatments [9–13].

Increased mitochondrial functions are important for tumorigenesis and the progression of OS [14, 15]. Levels of oxidative phosphorylation (OXPHOS) and ATP contents were elevated in OS cells after co-culturing with mesenchymal stem cells (MSCs), causing the more aggressive behaviors of OS cells [16]. Day et al. found that Bcl-xL stimulated OXPHOS in OS cells and inhibited cell apoptosis [17]. Our group has recently shown that POLRMT (RNA polymerase mitochondrial), a key mitochondrial protein for mitochondrial DNA (mtDNA) transcription, is overexpressed in human OS, promoting OS cell growth [18].

The biogenesis of the mitochondrial inner membrane in mammalian cells is dependent on distinct protein complexes. The formation of the translocase of inner mitochondrial membrane 8a (TIMM8a)–TIMM13 complex in the mitochondrial intermembrane space will promote the import and assembly of TIMM23 and other mitochondrial inner membrane proteins, including citrin and aralar1 [19–21]. This is essential for the integrity and functions of mitochondria. Defects in the assemble of the TIMM8a–TIMM13 complex could result in Mohr–Tranebjaerg syndrome (MTS), a rare neurodegenerative disease that is characterized by hearing loss and dystonia [19, 21, 22]. In the present study, we will show that TIMM13 is overexpressed in OS, required for OS cell growth in vitro and in vivo.

## Materials and methods

### Chemicals, reagents, and antibodies

The TIMM13 antibody (PA5-100352) and TIMM8a antibody (11179-1-AP) were purchased from Thermo-Fisher Invitrogen (Beijing, China). Cell culture reagents were described previously [10]. LY294002, ATP, N-acetyl cysteine (NAC), z-DEVD-fmk, and z-VAD-fmk were from Sigma (St. Louis, Mo). Cell counting kit -8 (CCK-8) was from Dojindo (Kumamoto, Japan). Fluorescence dyes, including EdU (5-Ethynyl-2′-deoxyuridine), DAPI (4′,6-diamidino-2-phenylindole), CellROX, TUNEL (Terminal deoxynucleotidyl transferase dUTP nick end labeling) and JC-1 (tetraethylbenzimidazolylcarbocyanine iodide), were purchased from Thermo-Fisher Invitrogen. All other antibodies were obtained from Cell Signaling Tech (Shanghai, China).

### Human tissues and cells

The surgery-resected OS tissues from twelve primary OS patients and the fragmented bone tissues removed from fracture surgeries of age- and gender-matched healthy donors were obtained from the author’s institution. MG63 and SaOS-2 immortalized cell lines [23], the primary human OS cells (pOS-1 and pOS-2, derived from primary patients), as well as the primary human osteoblasts, were cultivated through the described protocols [18]. hFOB1.19 human osteoblastic cells were from Dr. Liang and cells were cultured as described [24, 25]. The written informed consent was obtained from each participant. The protocols for using human tissues and cells were approved by the Ethics Committee of The First Affiliated Hospital of Zhengzhou University, according to the principles of the Declaration of Helsinki. To extract proteins from bone tissues, the fresh bone tissues were kept in cold PBS and washed thoroughly. The periosteum tissues, blood vessels, and fibrous tissues around the bones were carefully removed. The bone tissues were then cut into small pieces and placed in a mortar filled with liquid nitrogen. Tissues were then ground into powers before liquid nitrogen was volatilized. The powers were then dissolved into tissue lysis buffer (Biyuntian, Wuxi, China) at 100 mg powers in 250 μL lysis buffer, for 30 min on an ice bath. The mix was vortexed for 20 s every 5 min. Next, the mix was centrifuged at 12,000 rpm for 15 min. The supernatant containing the total protein of bone tissues was then transferred to a new centrifuge tube.

### TIMM13 and HOXC13 (Homeobox C13) silencing or overexpression

The lentiviral GV248 constructs encoding two different shRNAs against human *TIMM13*, shTIMM13-S1, and shTIMM13-S2 (with non-overlapping sequence), or two different shRNAs against human *HOXC13*, shHOXC13-S1 and shHOXC13-S2, GV248 construct encoding human *TIMM13* cDNA [NM_012458.4] or human HOXC13 cDNA ([NM_017410.3]) were provided by Genechem (Shanghai, China). The constructs were individually transfected to HEK-293T cells along with the lentivirus helper plasmids. The lentiviral particles were generated and enriched, filtered, and quantified. Infection of the viral particles and stable cell selection were described previously [18].

### TIMM13 knockout (KO)

As described previously [18], OS cells were first stably transduced with a LentiCas9-puro construct (Genechem) and were further transfected with Lenti-CRISPR-TIMM13-sgRNA-puro construct for 24 h. Stable cells were then selected by puromycin and were distributed into 96-well plates. Following *TIMM13* KO screening the TIMM13 KO OS cells were formed.

### Thiobarbituric acid reactive substance (TBAR) assay

TBAR activity in cellular or tissue lysates (30 lysate proteins of each sample) was measured through a commercial TBAR kit (MyBioSource, Beijing, China). The kit colorimetrically quantified the lipid peroxidation level and malondialdehyde (MDA) contents at 450 nm (with the reference of 595 nm).

### Cell counting kit-8 (CCK-8) assay

The genetically-modulated cells were placed into the 96-well plates at 3 × 10^3^ cells/200 μL/well. Cells were then maintained under 5% CO_2_, 37 °C incubator for 96 h. Afterward, 20 μL CCK-8 solution was added to each well and incubated for another 2 h. The CCK-8’s absorbance at 490 nm was measured by a microplate reader.

### Cell fluorescence experiments

The genetically-modulated cells were placed into 24-well plates at 5 × 10^4^ cells/500 μL/well and were then incubated for designated hours. Cells were then fixed with 4% paraformaldehyde for 30 min and then washed with PBS for 5 min, followed by incubating with 0.3% Triton at room temperature for 10 min. Afterward, the applied fluorescence dyes were added to the cells. Cells were then washed and visualized under a Leica microscope. The intensity of the fluorescence was quantified through the attached Leica software.

### Single-stranded DNA (ssDNA) ELISA

The genetically-modulated cells were placed into 24-well plates at 5 × 10^4^ cells/500 μL/well. Cells were then incubated for 48 h. Cell lysates (25 μg per treatment) were analyzed by an ssDNA ELISA kit (Roche Diagnostics, Shanghai, China). The ssDNA ELISA absorbance was tested at 450 nm.

### Transwell assays

Cell migration assays were performed by using Transwell chambers (Corning, NY, USA). In brief, the chambers were gently inserted into a 24-well plate and 600 µL of cell culture medium containing 15% fetal bovine serum (FBS) and 1% penicillin/streptomycin was added into the lower chamber. Cells were resuspended in the cell culture medium without FBS, 200 µL of which (2 × 10^4^ cells per chamber) were added to the upper surface of the chamber. After 24 h, cells were fixed with methanol for 10 min. The cells located on the upper surfaces were scraped out with cotton swabs. Cells located on the lower surface were stained with crystal violet and were photographed under a microscope. For invasion assays, Matrigel solution (BD Biosciences, San Jose, CA) was coated to the surface of the chamber.

### Gene and protein detection

The detailed protocols of quantitative Reverse Transcription PCR (qRT-PCR) and Western blotting were described in our previous study [18]. The isolation of the mitochondrial lysates was through a mitochondria-isolate kit (Sigma-Millipore) using high-speed centrifugation, and mitochondrial-null lysates was obtained as well. All the primers were designed, verified, and provided by Origene (Beijing, China). The uncropped blotting images were presented in Fig. S1.

### Constitutively-active mutant Akt1

A recombinant adenoviral constitutively-active Akt1 (caAkt1, S473D) construct was provided by Dr. Xu [26] and transduced to the described OS cells. Cells were then distributed to 96-well plates, and stable cells expressing caAkt1 were verified using Western blotting assays.

### Chromatin immunoprecipitation (ChIP) assay

As described [27] cell lysates were first homogenized by the MisonixSonicator 3000 Homogenizer [28] and were diluted in ChIP dilution buffer. Lysates were further immunoprecipitated with an anti-HOXC13 ((ab168368), Abcam) antibody, and the HOXC13-bound DNA was eluted by protein A/G agarose (Santa Cruz Biotech, Santa Cruz, CA), and NaCl was included. DNA containing the proposed *TIMM13* promoter site [29] was analyzed via quantitative PCR (qPCR).

*Other assays*, including mitochondrial depolarization (JC-1 staining assay), Caspase-3 activity assay, and Annexin V-PI FACS were described in detail in our previous study [18]. The detailed protocols of reactive oxygen species (ROS) detection (CellROX staining) and ATP contents detection were described elsewhere [30].

### Animal studies

The nude mice were described in detail in our previous study [18]. For in situ OS model, the pOS-1 cells (at four million cells per mouse) were injected into the proximal tibia of the nude mice [31]. After 4 weeks (28 days), in situ tumors were visualized via X-ray, and tumor volumes were measured. The animal protocols were according to the regulations of the institutional animal care and use committee (IACUC) and with approval from Zhengzhou University.

### Tissue immuno-fluorescence assays

Briefly, the 4% paraformaldehyde-fixed, paraffin-embedded tissue sections were examined. After dewaxing, the tissue slices were incubated with Protease K for 30 min at 37 °C and were washed with PBS twice. The equilibration buffer was then utilized to cover the tissue slices at room temperature. After 30 min, TUNEL buffer was used to cover the slices for 1 h at 37 °C. Next, slices were washed with PBS and stained with DAPI. The slices were then photographed under a Leica confocal microscope.

### Statistical analyses

In vitro, experiments were repeated five times. Data in this study were with normal distribution and were always presented as mean ± standard deviation (SD). Statistical differences were analyzed by one-way ANOVA with post hoc Bonferroni test (SPSS version 20.0, SPSS Co., Chicago, CA) for multiple groups. The two-tailed unpaired *t*-test was applied for the comparison between the two groups. *P* < 0.05 was considered statistically significant.

## Results

### Mitochondrial TIMM13 upregulation in OS tissues and cells

**Fig. 1:**
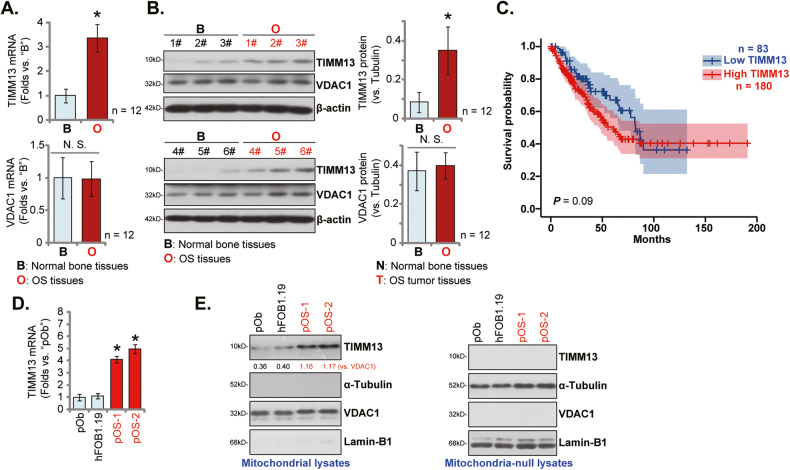
Mitochondrial TIMM13 upregulation in OS tissues and cells. Expression of listed mRNAs (**A**) and proteins (**B**) in the described OS tissues (“O”) and the fragmented bone tissues (“B”) removed from fracture surgeries of age and gender-matched healthy donors was shown, with results quantified. TCGA database shows the Kaplan–Meier survival results of *TIMM13*-high sarcoma patients and *TIMM13*-low sarcoma patients (**C**). Expression of *TIMM13* mRNA and TIMM13 protein (in both mitochondria lysates and mitochondria-null lysates) in primary human OS cells (pOS-1 and pOS-2), primary human osteoblasts (“pOb”) and hFOB1.19 osteoblasts were shown (**D**, **E**). Error bars stand for mean ± standard deviation (SD). *P < 0.05 versus “B” tissues/“pOb” cells.

First, TIMM13 expression in human OS tissues was analyzed. The surgery-resected OS tissues (“O”) from twelve (*n* = 12) primary OS patients and the fragmented bone tissues (“B”) removed from fracture surgeries of age and gender-matched healthy donors were obtained and analyzed. Figure 1A demonstrated that *TIMM13* mRNA in the OS tissues was significantly higher (over threefold) than that in normal bone tissues (*P* < 0.05). Testing TIMM13 protein expression, through Western blotting assays, showed that TIMM13 protein levels were elevated in six representative primary OS tissues (derived from Patient-1# to Patient-6#) (Fig. 1B). All twelve sets of blotting data were combined and analyzed, and results demonstrated that TIMM13 protein upregulation in OS tissues was significant (*P* < 0.05 versus normal bone tissues, Fig. 1B). The mRNA and protein expression of the mitochondrial protein VDAC1 was indifferent between the two groups of tissues (Fig. 1A, B). The Cancer Genome Atlas (TCGA) database was consulted next and Kaplan–Meier survival results revealed that high *TIMM13* expression in sarcoma patients tended to have a poor prognosis (*P* = 0.09) (Fig. 1C).

TIMM13 expression in different human OS cells was examined next. As shown, *TIMM13* mRNA (Fig. 1D) and TIMM13 protein (in the mitochondrial lysates, Fig. 1E) levels in the primary human OS cells (pOS-1 and pOS-2, see our previous study [18]) were significantly higher than those in the primary human osteoblasts (“Ob”) and osteoblastic OB-6 cells (Fig. 1D, E). *TIMM13* protein was undetected in mitochondria-null lysates (Fig. 1E). These results confirmed mitochondrial TIMM13 upregulation in OS tissues and cells.

### TIMM13 is crucial for mitochondrial functions in OS cells

**Fig. 2:**
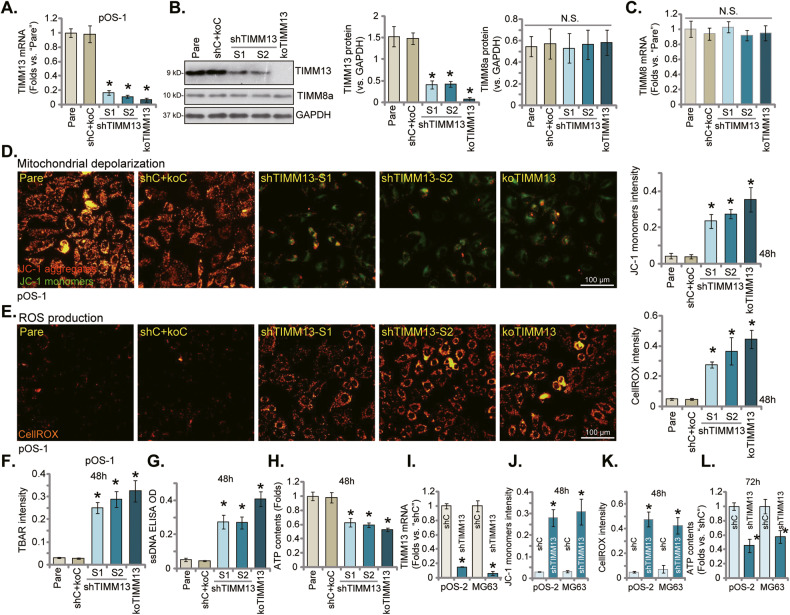
TIMM13 is crucial for mitochondrial functions in OS cells. The primary OS cells, pOS-1, expressing the TIMM13 shRNA (“shTIMM13-S1”/“shTIMM13-S2”, with different sequences), the CRISPR/Cas9–TIMM13–KO plasmid (“koTIMM13”), or the scramble control shRNA plus the Cas9 empty vector (“shC+koC”), were established. Expression of listed mRNAs and proteins was shown (**A**–**C**); cells were further cultivated under the complete medium for the designated hours, mitochondrial depolarization was examined by JC-1 staining assays (**D**); ROS production was tested by CellROX assay (**E**), with lipid peroxidation examined by TBAR assays (**F**). The cellular single-stranded DNA (ssDNA) contents (**G**) and ATP levels (**H**) were examined as well. The primary OS cells, pOS-2, or the immortalized MG63 cells were infected with lentiviral shTIMM13-S1 (“shTIMM13”) or the lentiviral scramble control shRNA (“shC”), and stable cells were formed following puromycin selection. *TIMM13* mRNA expression was tested by qRT-PCR assays (**I**). Cells were further cultivated under the complete medium for the designated hours, JC-1 dye assays were carried out for testing mitochondrial depolarization (**J**); ROS levels were examined through CellROX (**K**) assays, with the ATP contents examined as well (**L**). “Pare” stands for the parental control of OS cells. Error bars stand for mean ± standard deviation (SD, *n* = 5). *P < 0.05 versus “Pare”/“shC” cells. Experiments in this figure were repeated five times. Scale bar = 100 μm.

TIMM13 is a mitochondrial protein localized in the inter-membrane space [19, 21]. We first examined whether TIMM13 is important for maintaining mitochondrial functions in human OS cells. Genetic strategies, as described in our previous study [18], were utilized. The primary human osteosarcoma cells, pOS-1 [18], were infected with the lentiviral particles with shRNAs targeting human *TIMM13*, namely shTIMM13-S1 or shTIMM13-S2 (with non-overlapping sequences). Stable pOS-1 cells were then formed after further culturing cells in the puromycin selection complete medium. Alternatively, a CRISPR/Cas9–TIMM13–KO plasmid was established and was transduced to Cas9-expressing pOS-1 cells. Through *TIMM13* KO screening, the TIMM13 KO pOS-1 cells were formed: namely “koTIMM13” cells. The control pOS-1 cells were constructed with the lentiviral scramble control shRNA plus Cas9 control construct (“shC+koC”). To verify the efficiency of these genetic treatments, qRT-PCR assay results in Fig. 2A demonstrated that *TIMM13* mRNA levels were dramatically decreased in the shTIMM13 pOS-1 cells and the koTIMM13 pOS-1 cells (*P* < 0.05 vs. “shC+koC” cells). The TIMM13 protein expression, tested by Western blotting assays, was downregulated as well (Fig. 2B). On contrast, *TIMM8a* mRNA (Fig. 2C) and protein (Fig. 2B) expression was unchanged by the applied TIMM13 shRNA and the CRISPR/Cas9 KO construct.

Significantly, TIMM13 shRNA or KO in pOS-1 cells decreased mitochondrial membrane potential (∆Ψ) and induced mitochondrial depolarization, as the JC-1 fluorescence changed from orange (JC-1 aggregates) to green (JC-1 monomers) (Fig. 2D) [32–35]. Moreover, TIMM13 silencing or depletion in pOS-1 cells induced mitochondrial ROS production and oxidative injury, evidenced by the increased intensity of the CellROX orange fluorescence (Fig. 2E). The thiobarbituric acid reactive substances (TBAR) fluorometric assay kit was utilized. Results showed that the TBAR intensity was significantly increased in pOS-1 cells with TIMM13 shRNA or KO (Fig. 2F). Furthermore, the increased single-stranded DNA (ssDNA) contents implied DNA breaks accumulation following TIMM13 shRNA or KO in pOS-1 cells (Fig. 2G). The cellular ATP contents were decreased with TIMM13 depletion (Fig. 2H). These results implied that TIMM13 silencing/depletion led to mitochondrial dysfunction in primary OS cells, causing mitochondrial depolarization, ROS production, and oxidative injury as well as lipid peroxidation, DNA breaks, and ATP depletion.

To the primary OS cells derived from another patient, pOS-2, as well as immortalized MG-63 cells, the shTIMM13-S1 lentiviral particles were added. Stable cells were again selected by adding puromycin and these cells were named as “shTIMM13” cells. Control cells were infected with the lentiviral scramble control shRNA (“shC”). The shTIMM13-S1 treatment resulted in profound *TIMM13* mRNA silencing in the primary and MG-63 cells (Fig. 2I). In the OS cells, shTIMM13 induced depolarization of mitochondria (accumulation of JC-1 green monomers, Fig. 2J) and ROS production (CellROX intensity increasing, Fig. 2K). The cellular ATP contents were reduced in TIMM13-silenced OS cells (Fig. 2L). In SaOS-2 cells TIMM13 silencing by shTIMM13-S1 (“shTIMM13”, Fig. S2A) also induced mitochondrial depolarization (Fig. S2B) and ROS production (Fig. S2C). Together, these results showed that TIMM13 is crucial for maintaining the mitochondrial functions in OS cells. Whereas TIMM13 silencing or depletion significantly impaired mitochondrial functions.

### TIMM13 depletion provokes mitochondrial apoptosis cascade in OS cells

**Fig. 3:**
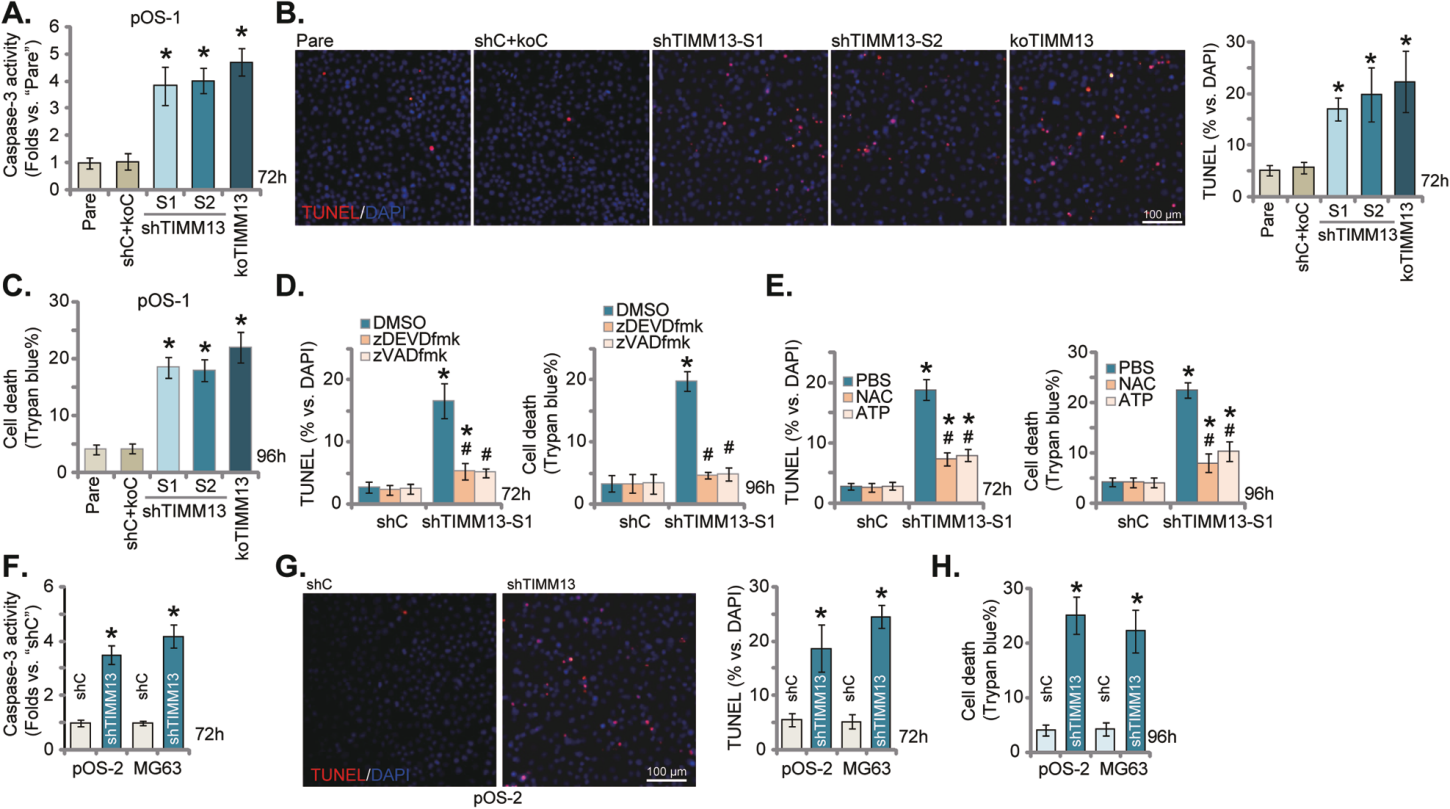
TIMM13 depletion provokes mitochondrial apoptosis cascade in OS cells. The primary OS cells, pOS-1, expressing the TIMM13 shRNA (“shTIMM13-S1”/“shTIMM13-S2”, with different sequences), the CRISPR/Cas9–TIMM13–KO plasmid (“koTIMM13”), or the scramble control shRNA plus the Cas9 empty vector (“shC+koC”), were established and cultivated under the complete medium for designated hours, the relative caspase-3 activity (**A**) was examined; cell apoptosis was tested by TUNEL staining assay (**B**); cell death was quantified through Trypan blue staining assays (**C**). pOS-1 cells with shTIMM13-S1 or shC were treated with zVAD-fmk (40 μM), zDEVD-fmk (40 μM), NAC (400 μM) or ATP (1 mM), and cells were cultivated under the complete medium for designated hours; cell apoptosis and death were tested by TUNEL staining and Trypan blue staining assays (**D**, **E**). The primary OS cells, pOS-2, or the immortalized MG63 cells, with shTIMM13-S1 (“shTIMM13”) or the scramble control shRNA (“shC”), were cultivated under the complete medium for designated hours; The relative caspase-3 activity (**F**), cell apoptosis (by measuring TUNEL-positive nuclei percentage, **G**) and cell death (Trypan blue ratio, **H**) were tested. “Pare” stands for the parental control OS cells. Error bars stand for mean ± standard deviation (SD, *n* = 5). *P < 0.05 versus “Pare”/“shC” cells. ^#^*P* < 0.05 versus “DMSO”/“PBS” (**D**, **E**). Experiments in this figure were repeated five times. Scale bar = 100 μm.

In OS cells and other human cancer cells following mitochondrial dysfunction, the apoptosis cascade will be activated [36–38]. As TIMM13 shRNA/KO disrupted mitochondrial functions in primary and immortalized OS cells, we tested whether it can provoke apoptosis. As shown in Fig. 3A, the caspase-3 activity was significantly increased in pOS-1 cells bearing the TIMM13 shRNA or the CRISPR/Cas9–TIMM13–KO construct. Supporting apoptosis activation, we found that the percentage of TUNEL positively-stained nuclei was significantly increased in TIMM13-silenced and TIMM13-KO pOS-1 cells (Fig. 3B). Evidenced by the increased trypan blue staining, we found that TIMM13 knockdown/KO induced moderate but significant cell death in pOS-1 cells (Fig. 3C).

To block apoptosis activation, two caspase inhibitors were applied, including the caspase-3 specific inhibitor z-DEVD-fmk and the pan-caspase inhibitor z-VAD-fmk. The two almost blocked apoptosis and cell death by shTIMM13-1 (Fig. 3D). Supplement with the antioxidant NAC or ATP ameliorated shTIMM13-S1-induced apoptosis (TUNEL assays) and cell death (Trypan blue assays) (Fig. 3E). These results implied that ROS production and ATP depletion should be the important trigger of TIMM13 silencing-induced cytotoxicity in OS cells. In pOS-2 primary cells and immortalized MG-63 cells, TIMM13 silencing by the shTIMM13-S1 increased caspase-3 activity (Fig. 3F) and TUNEL positively-stained nuclei percentage (Fig. 3G). TIMM13 silencing also induced the death of the OS cells, evidenced by increased Trypan blue staining (Fig. 3H). TIMM13 shRNA also induced apoptosis in SaOS-2 cells and increased TUNEL nuclei percentage (Fig. S2D). Together TIMM13 depletion provoked a mitochondrial apoptosis cascade and induced death in different OS cells.

### TIMM13 depletion inhibits OS cell proliferation and migration

**Fig. 4:**
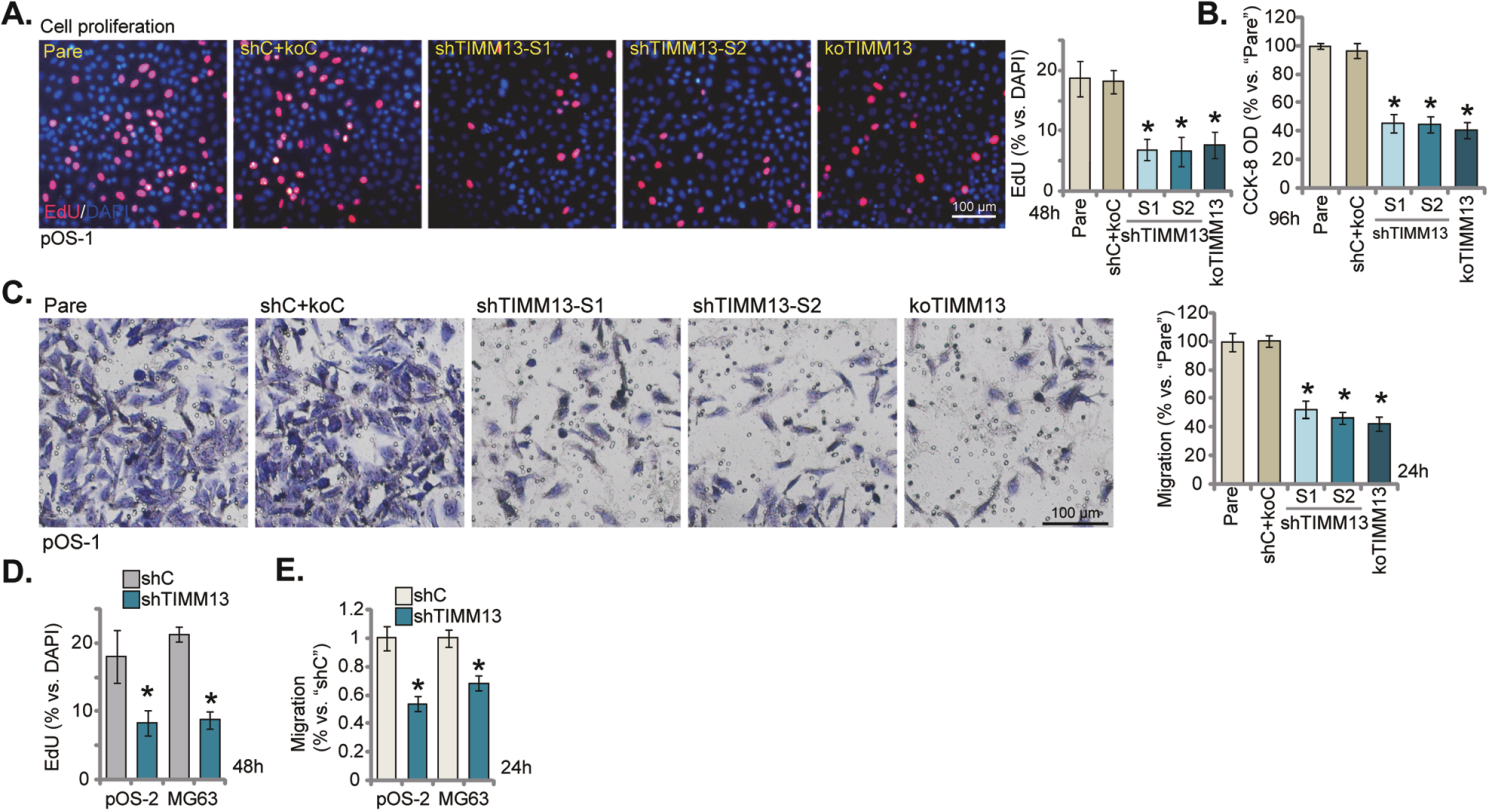
TIMM13 depletion inhibits OS cell proliferation and migration. The primary OS cells, pOS-1, expressing the TIMM13 shRNA (“shTIMM13-S1”/“shTIMM13-S2”, with different sequences), the CRISPR/Cas9–TIMM13–KO plasmid (“koTIMM13”), or the scramble control shRNA plus the Cas9 empty vector (“shC+koC”), were established and cultivated under the complete medium for designated hours; cell proliferation, viability, and migration were examined by EdU staining (**A**), CCK-8 (**B**), and “Transwell” (**C**) assays, respectively. The primary OS cells, pOS-2, or the immortalized MG63 cells, with shTIMM13-S1 (“shTIMM13”) or the scramble control shRNA (“shC”), were cultivated under the complete medium for designated hours; cell proliferation (**D**) and migration (**E**) were tested similarly, with results quantified. “Pare” stands for the parental control of OS cells. Error bars stand for mean ± standard deviation (SD, *n* = 5). *P < 0.05 versus “Pare”/“shC” cells. Experiments in this figure were repeated five times. Scale bar = 100 μm.

Increased mitochondrial biogenesis and energy metabolism are vital for cancer cell growth [14, 15]. Since TIMM13 is important for mitochondrial biogenesis and ATP production [19, 21, 22], we tested whether TIMM13 depletion could affect OS cell growth. As shown, in pOS-1 primary cells TIMM13 silencing, by shTIMM13-S1 and shTIMM13-S2, or CRISPR/Cas9-induced TIMM13 KO, largely decreased EdU-positive nuclei percentage (Fig. 4A), suggesting that TIMM13 depletion inhibited OS cell proliferation (Fig. 4A). In addition, pOS-1 cell viability (CCK-8 OD, Fig. 4B) was significantly decreased after TIMM13 silencing or depletion. Furthermore, TIMM13 shRNA or KO suppressed pOS-1 cell in vitro migration (Fig. 4C). In pOS-2 and immortalized MG-63 cells, TIMM13 silencing, by the shTIMM13-S1, inhibited cell proliferation (EdU nuclei ratio decreasing, Fig. 4D) and in vitro migration (Fig. 4E) as well. TIMM13 silencing also inhibited proliferation (Fig. S2E) and migration (Fig. S2F) in SaOS-2 cells.

### Ectopic TIMM13 overexpression further promotes OS cell proliferation and migration

**Fig. 5:**
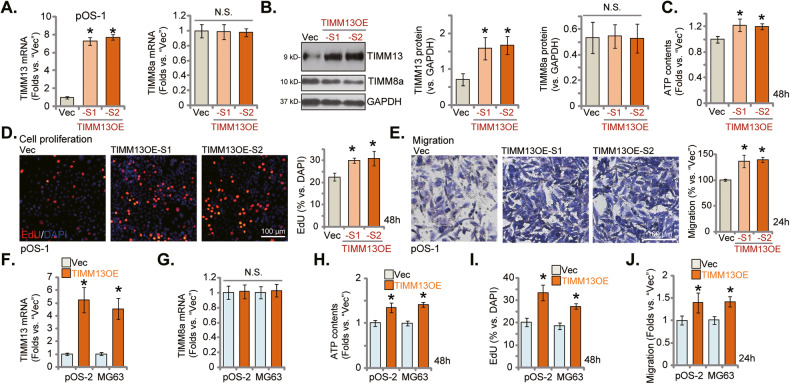
Ectopic TIMM13 overexpression further promotes OS cell proliferation and migration. pOS-1 primary cells, expressing the GV248-TIMM13-GFP-expressing construct (TIMM13OE-S1/S2, two stable selections) or the GV248 empty vector (“Vec”), were established, and expression of listed mRNAs and proteins was shown (**A**, **B**), with the cellular ATP contents examined (**C**). Cell proliferation and migration were examined by EdU staining (**D**) and “Transwell” (**E**) assays, respectively. The primary OS cells, pOS-2, or the immortalized MG63 cells, with TIMM13-expressing construct (“TIMM13OE”) or the empty vector (“Vec”), were established, *TIMM13* and *TIMM8a* mRNA expression were tested by qRT-PCR assays (**F**, **G**); cells were further cultivated under the complete medium for designated hours; ATP contents (**H**), cell proliferation (by measuring EdU-positive nuclei ratio, **I** and migration (by measuring migrated cell number, **J** were tested similarly. Error bars stand for mean ± standard deviation (SD, *n* = 5). *P < 0.05 vs. “Vec” cells. Experiments in this figure were repeated five times. Scale bar = 100 μm (**D**, **E**).

We next hypothesized that ectopic overexpression of TIMM13 might possibly further promote OS cell growth. Therefore, the lentiviral particles containing the GV248-TIMM13-GFP-expressing construct were added to pOS-1 primary cells. Following selection by puromycin, two stable selections, TIMM13OE-S1 (selection 1) and TIMM13OE-S2 (selection s), were formed. As demonstrated, *TIMM13* mRNA levels were significantly increased in TIMM13OE pOS-1 cells (Fig. 5A), whereas *TIMM8a* mRNA levels were unchanged (Fig. 5A). Protein expression of TIMM13, but not TIMM8a, was elevated as well in TIMM13OE-S1 and TIMM13OE-S2 pOS-1 cells (Fig. 5B). As shown, ATP contents were significantly increased in TIMM13OE pOS-1 cells (Fig. 5C). Functional studies demonstrated that TIMM13 ectopic overexpression augmented pOS-1 cell proliferation (EdU positively-stained nuclei ratio increasing, Fig. 5D) and in vitro cell migration (Fig. 5E). These results further supported an oncogenic role of TIMM13 in OS.

The TIMM13-expressing construct was also transfected to pOS-2 primary cells and immortalized MG-63 cells and stable cells established after puromycin selection, namely TIMM13OE cells. Compared to OS cells with the empty vector (“Vec”), *TIMM13* mRNA levels were dramatically increased in the TIMM13OE OS cells (Fig. 5F), where *TIMM8a* mRNA expression was unchanged (Fig. 5G). Levels of intracellular ATP were increased in the TIMM13OE OS cells (Fig. 5H). Moreover, ectopic overexpression of TIMM13 further enhanced cell proliferation (EdU assays, Fig. 5I) and migration (Fig. 5J) in the primary and immortalized OS cells.

### TIMM13 is important for Akt-mTOR activation in primary human OS cells

**Fig. 6:**
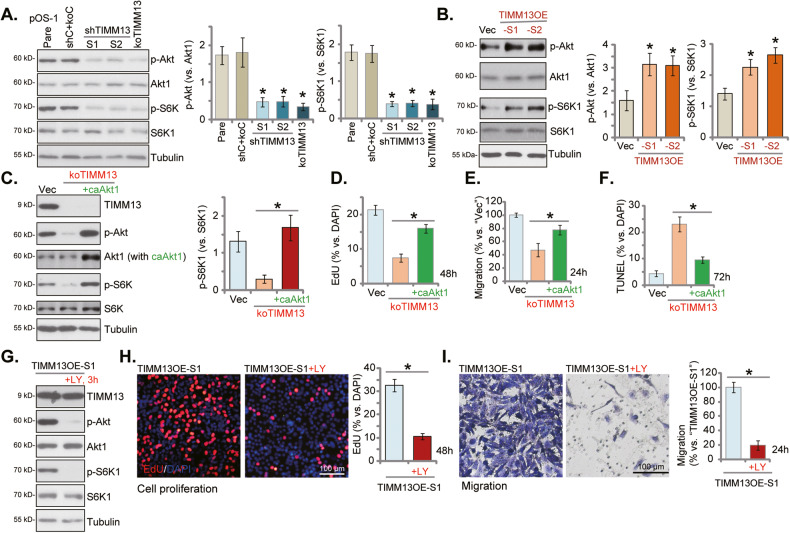
TIMM13 is important for Akt-mTOR activation in primary human OS cells. The primary OS cells, pOS-1, expressing the TIMM13 shRNA (“shTIMM13-S1”/“shTIMM13-S2”, with different sequences), the CRISPR/Cas9–TIMM13–KO construct (“koTIMM13”), or the scramble control shRNA plus the Cas9 empty vector (“shC+koC”), were established; Expression of listed proteins was shown (**A**). The primary pOS-1 cells, expressing the GV248-TIMM13-GFP-expressing construct (TIMM13OE-S1/S2, two stable selections) or the GV248 empty vector (“Vec”) were established, and expression of listed proteins was shown (**B**). The koTIMM13 pOS-1 cells were transduced with adenovirus-packed constitutively-active Akt1 (caAkt1, S473D), and stable cells were established. Expression of listed proteins was shown (**C**); cells were further cultivated under the complete medium for designated hours, and cell proliferation, migration, and apoptosis were examined by EdU staining (**D**), “Transwell” (**E**), and TUNEL staining (**F**) assays, respectively, with results quantified. TIMM13OE-S1 cells were treated with or without LY294002 (“LY”, 250 nM) for designated hours, expression of listed proteins was shown (**G**); cell proliferation (**H**) and migration (**I**) were tested. Error bars stand for mean ± standard deviation (SD, n = 5). *P < 0.05 vs. “shC+koC”/“Vec” cells. *P < 0.05 (**C**–**I**). Experiments in this figure were repeated five times. Scale bar = 100 μm.

Disrupting mitochondrial functions by TIMM13 depletion caused ATP reduction and energy shortage, which could cause the inactivation of pro-cancerous cascades. The Akt-mTOR cascade is one key cascade required for OS tumorigenesis and progression [39–43]. We, therefore, analyzed the potential effect of TIMM13 on Akt-mTOR activation in OS cells. As shown, in pOS-1 primary cells TIMM13 shRNA or KO (see Figs. 2–4) largely inhibited phosphorylation of Akt and S6K1 (Fig. 6A), indicating Akt-mTOR inactivation. Conversely, as shown in Fig. 6B, Akt-mTOR activation was augmented in TIMM13-overexpressed pOS-1 cells (TIMM13OE-S1 and TIMM13OE-S2, see Fig. 5). These results implied that TIMM13 is indeed important for Akt-mTOR cascade activation in OS cells.

Next, the constitutively-active Akt1 (caAkt1, S473D)-expressing adenovirus [26, 44] was transduced to koTIMM13 pOS-1 cells, restoring Akt and S6K1 phosphorylation (Fig. 6C). Importantly, TIMM13 KO-induced proliferation inhibition (EdU-ratio decreasing, Fig. 6D), migration reduction (Fig. 6E) and apoptosis (TUNEL ratio increasing, Fig. 6F) were largely ameliorated by caAkt1. These results implied that Akt-mTOR inhibition participated in TIMM13 depletion-induced anti-OS cell activity.

To further support our hypothesis, in TIMM13-overexpressed pOS-1 cells (“TIMM13OE-S1”, see Fig. 5), the pan PI3K-Akt-mTOR inhibitor LY294002 was added [45]. LY294002 blocked Akt and S6K1 phosphorylation in pOS-1 cells, without affecting TIMM13 expression (Fig. 6G). Importantly, LY294002 potently inhibited pOS-1 cell proliferation (Fig. 6H) and migration (Fig. 6I) in the TIMM13OE-S1 pOS-1cells. Therefore, TIMM13 overexpression-facilitated pOS-1 cell proliferation and migration were largely inhibited by LY294002, further supporting that activating the Akt-mTOR cascade is important for TIMM13-driven OS cell progression in vitro.

### HOXC13-dependent TIMM13 transcription is increased in OS tissues and cells

**Fig. 7:**
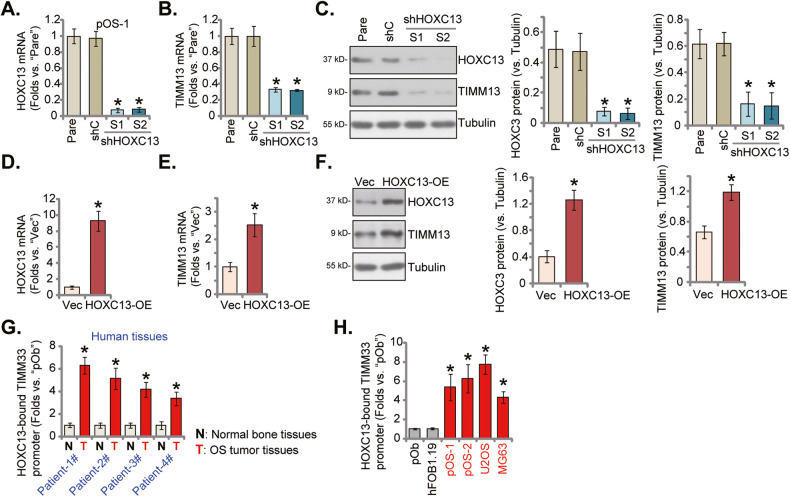
HOXC13-dependent TIMM13 transcription is increased in OS tissues and cells. The primary pOS-1 cells expressing the HOXC13 shRNA (“sh HOXC13-S1”/“shHOXC13-S2”, with different sequences), the scramble control shRNA (“shC”), the TIMM13-expressing construct (“HOXC13-OE”) or the empty vector (“Vec”), were established, and expression of listed genes and proteins were shown (**A**–**F**). Chromosome IP (ChIP) results showed the relative levels of HOXC13-bound *TIMM13* promoter in the listed human OS tumor tissues and pare-tumor normal bone tissues (**G**), as well as in the listed OS cells and osteoblasts (**H**). “Pare” stands for the parental control of OS cells. Error bars stand for mean ± standard deviation (SD, *n* = 5). * *P* < 0.05 vs. “Pare”/“shC” cells. Experiments in this figure were repeated five times.

We also explored the underlying mechanism of TIMM13 upregulation in OS. Since *TIMM13* mRNA and protein levels were both upregulated, we focused on the transcriptional mechanism. An early study has verified that HOXC13 should be an important transcription factor of *TIMM13* in human asynchronous T98G cells [29]. A set of two different lentiviral HOXC13 shRNA, with non-overlapping shRNA sequences (shHOXC13-S1 and shHOXC13-S2), were individually transduced to pOS-1 cells, and stable cells established after selection. Each of the applied shRNA resulted in robust *HOXC13* mRNA silencing in pOS-1 cells (Fig. 7A), and significantly downregulated *TIMM13* mRNA (Fig. 7B). HOXC13 and TIMM13 protein levels were both decreased in shHOXC13-expressing pOS-1 cells (Fig. 7C).

To increase HOXC13 expression a lentiviral overexpression construct was stably transduced to pOS-1 cells, and stable cells (“HOXC13-OE”) were again formed after selection. *HOXC13* mRNA expression in HOXC13-OE cells was over 8–10 times higher than that in the vector control cells (Fig. 7D). HOXC13 OE increased *TIMM13* transcription and mRNA expression (Fig. 7E) in pOS-1 cells. HOXC13 and TIMM13 protein expression was significantly increased in HOXC13-OE cells as well (Fig. 7F). These silencing and overexpression results supported that HOXC13 could be an important transcription factor of TIMM13 in primary human OS cells.

Importantly, in the OS tissues of four representative patients (Patient-1# to Patient 4#, see Fig. 1), ChIP assay results demonstrated that HOXC13 binding to the *TIMM13* promoter was significantly higher than that in the matched normal bone tissues (Fig. 7G). Moreover, HOXC13 binding to the *TIMM13* promoter was significantly elevated in different primary/established OS cells (Fig. 7H), when compared to the human osteoblasts (Fig. 7H). These results together supported that TIMM13 upregulation in OS could be due to increased binding between HOXC13 and *TIMM13* promoter.

### TIMM13 KO inhibits in situ OS xenograft growth

**Fig. 8:**
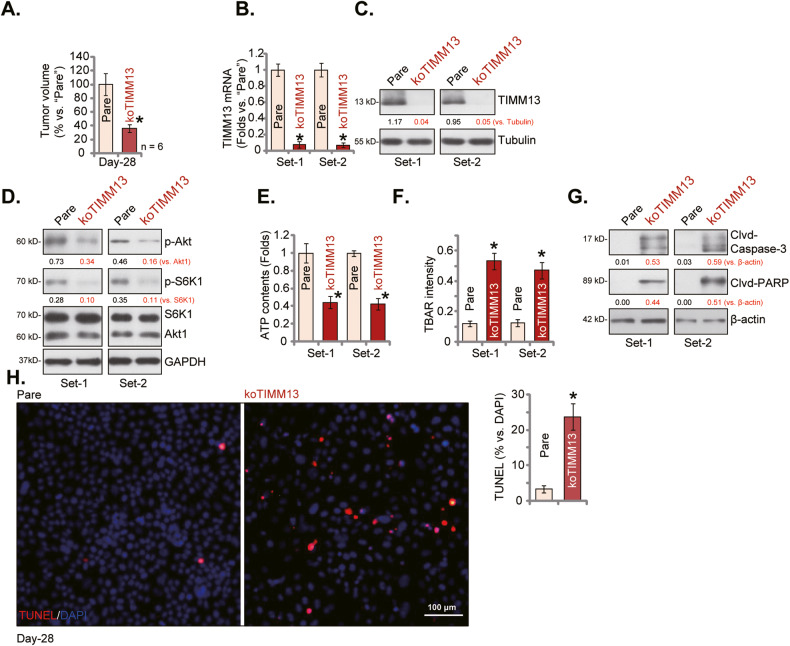
TIMM13 KO inhibits in situ OS xenograft growth. The parental control pOS-1 cells or the koTIMM13 pOS-1 cells were injected into the proximal tibia of the nude mice. After 28 days, in situ pOS-1 xenografts were formed and tumor volumes were measured under X-ray (**A**); expression of listed genes and proteins in the listed in situ tumor lysates was shown (**B**–**D**, **G**); ATP contents (**E**) and the TBAR activity (**F**) in the tumor lysates were tested as well. The representative TUNEL fluorescence images of in situ xenograft slides were presented as well, with TUNEL ratio quantified (**H**). Error bars stand for mean ± standard deviation (SD). Scale bar = 100 μm (**H**).

At last, the koTIMM13 pOS-1 cells or the parental control cells, at four million cells per mouse, were injected in situ into the proximal tibia of the nude mice. The in situ pOS-1 xenografts were formed within four weeks (“Day-28”) and the xenografts were visualized under X-ray and tumor volumes were measured. Results showed that the volumes of in situ koTIMM13 pOS-1 xenografts (“koTIMM13”) were significantly lower than those of parental control pOS-1 xenografts (“Pare”) (Fig. 8A). The in situ pOS-1 xenografts were then carefully resected through surgery, and two sets of tumors of both “Pare” and “koTIMM13” groups were analyzed for the signaling genes/proteins. As shown *TIMM13* mRNA (Fig. 8B) and protein (Fig. 8C) levels were robustly decreased in the two koTIMM13 in situ pOS-1 xenograft tissues, where Akt and S6K1 phosphorylations were decreased (Fig. 8D). ATP contents were significantly reduced in koTIMM13 in situ pOS-1 xenografts (Fig. 8E), and TBAR activity was increased (Fig. 8F). The cleaved-caspase-3 and cleaved-PARP levels were significantly increased in the in situ koTIMM13 pOS-1 tumor tissues (Fig. 8G). The tissue immuno-fluorescence assay results, Fig. 8H, demonstrated a significantly increased number of TUNLE-positive nuclei in the in situ koTIMM13 tumor slides. These results together supported that TIMM13 KO induced Akt-mTOR inactivation, ATP depletion, oxidative injury, and apoptosis in in situ OS tumors.

## Discussion

Molecularly-targeted therapies are the focus of the clinical and basic research of OS [43, 46–49]. Inhibitors of multiple signaling pathways, including mTOR, Src kinase family, and VEGFR, are under clinical evaluation and could improve outcomes in patients with relapsed or refractory OS [43, 46–49]. Duffaud et al., have evaluated the efficacy and safety of a multi-kinase inhibitor raccorafenib for the treatment of metastatic OS. Results showed that recurrent, progressive, metastatic OS patients with previous failure of conventional chemotherapy could benefit from raccorafenib, delaying disease progression [50]. In an independent double-blind clinical trial, the median PFS for the relapsed, refractory, or metastatic OS patients was 3.6 months in the recorafenib group and only 1.7 months in the placebo group [51].

Both the mitochondrial genome and the mitochondrial functions are dysregulated in OS, which is essential for tumorigenesis [14, 15]. Oxidative phosphorylation and ATP production were upregulated in OS cells co-culturing with mesenchymal stem cells, causing enhanced cell migration and other aggressive behaviors of OS cells [16]. Bcl-xL exerted an anti-apoptotic activity in OS cells by promoting OXPHOS and ATP production [17]. POLRMT dictates mitochondrial genome (mtDNA) transcription, thereby promoting oxidative phosphorylation and maintaining mitochondrial functions [52–55]. Our previous study has found that POLRMT is upregulated in OS tissues and cells. Whereas genetic depletion of POLRMT resulted in decreased mtDNA transcription, disrupted mitochondrial functions, and robust anti-OS cell activity [18]. These results clearly supported the role of mitochondrial functions in OS progression.

We here provided preclinical results supporting that TIMM13, a mitochondrial protein, could be a novel and promising therapeutic target of OS. Mitochondria-localized TIMM13 protein is upregulated in human OS. *TIMM13* mRNA and protein levels in the human OS tumor tissues were significantly higher than those in the matched adjacent normal bone tissues. Upregulation of *TIMM13* mRNA and protein was detected in various OS cells as well. Significantly, high *TIMM13* expression in sarcoma patients has a poor prognosis.

Studies have supported the oncogenic role of HOXC13 in different cancers. Dai et al. reported that overexpressed HOXC13 promoted cervical cancer proliferation, migration, and glycolysis possibly by activating β-catenin/c-Myc signaling cascades [56]. Li et al., revealed that HOXC13 expression is elevated in breast cancer, and it is associated with the prognosis of the patients [57]. Yao et al. showed that HOXC13 overexpression increased cyclin D1 and cyclin E1 expression to promote lung adenocarcinoma cell proliferation [58]. HOXC13 is a verified transcription factor of *TIMM13* in human cells [29]. We showed that TIMM13 transcription and expression were decreased following HOXC13 silencing, but were increased after HOXC13 overexpression in primary OS cells. Moreover, ChIP assay results confirmed that HOXC13-*TIMM13* promoter binding was increased in human OS tissues and cells. Therefore, the upregulation of TIMM13 in OS tissues and cells could be due to increased *TIMM13* transcriptional machinery through HOXC13.

Importantly, the elevated expression of mitochondrial TIMM13 is critical for maintaining mitochondrial functions in OS cells and is important for OS cell growth. TIMM13 shRNA or KO caused significant mitochondrial dysfunction, inducing mitochondrial depolarization, oxidative injury, lipid peroxidation, DNA damage, and ATP depletion, eventually causing apoptotic cell death. OS cell growth and migration were hindered by TIMM13 silencing/KO as well. Contrarily, ectopic overexpression of TIMM13 further increased ATP contents and augmented primary OS cell growth and migration. In vivo, TIMM13 KO potently inhibited in situ OS xenograft growth in the proximal tibia of nude mice. ATP depletion, oxidative injury, and apoptosis were detected in TIMM13 KO in situ OS tumors. Therefore, targeting the mitochondrial protein TIMM13 could be a novel strategy to inhibit OS.

Akt-mTOR cascade is often overactivated in OS, which is associated with tumorigenesis and development. Activation of this cascade is vital for OS cell proliferation, cell cycle progression, migration, and apoptosis inhibition, as well as metastasis and therapy resistance [41, 42, 59–63]. Blockage of this cascade by the small molecular inhibitors could result in robust anti-OS activity [41, 42, 59–63]. The underlying mechanisms of Akt-mTOR overactivation in OS are still elusive. Here we found that TIMM13 is important for the activation of Akt-mTOR in OS. Akt-S6K1 phosphorylation was decreased with TIMM13 shRNA/KO in primary OS cells but was increased in TIMM13-overexpressed OS cells. Akt-mTOR activation was also inhibited in TIMM13 KO in situ xenografts. Importantly, caAkt1 restored Akt-mTOR activation and largely attenuated TIMM13 KO-induced anti-OS cell activity. Moreover, an Akt-mTOR blocker LY294002 reversed the pro-OS cell activity by TIMM13 overexpression. Therefore TIMM13-driven OS cell growth is mediated, at least in part, by promoting Akt-mTOR activation.

Maintaining normal mitochondrial functions is important for Akt-mTOR activation [64], whereas mitochondrial inhibitors could result in ATP depletion and Akt-mTOR inhibition [64]. TIMM8a-TIMM13 association is crucial for mitochondrial inner membrane biogenesis. Contrarily, disruption of this complex could inhibit citrin and aralar1 expression, aspartate-glutamate carrier (AGC) function, mitochondrial protein import, and NADH shuttling, thereby causing significant mitochondrial dysfunction [21]. Moreover, disruption of TIMM13-TIMM8a association by genetic mutation could result in elongation and/or increased fusion of mitochondria [19, 22]. Therefore, it is possible that TIMM13 deletion resulted in abnormal mitochondrial function, causing ATP depletion and oxidative injury, which in turn led to Akt-mTOR inhibition. This could be a key mechanism to explain TIMM13-driven OS progression. However, the underlying molecular mechanisms need to be further explored.

## Conclusion

HOXC13-driven overexpression of the mitochondrial protein TIMM13 is important for OS cell growth, representing as a novel and promising therapeutic target.

## Supplementary information

SUPPLEMENTAL Figure 1

SUPPLEMENTAL Figure 2

aj-checklist Form

## Data Availability

All data are available upon request.
