# Supplementary material for: HOXC13-driven TIMM13 overexpression promotes osteosarcoma cell growth
Source: Cell Death Dis. 2023 Jul 5;14(7):398. doi: 10.1038/s41419-023-05910-0 (PMC10322838; doi:10.1038/s41419-023-05910-0)

# Figure S1: The uncropped blotting images of the study.

## Figure 1

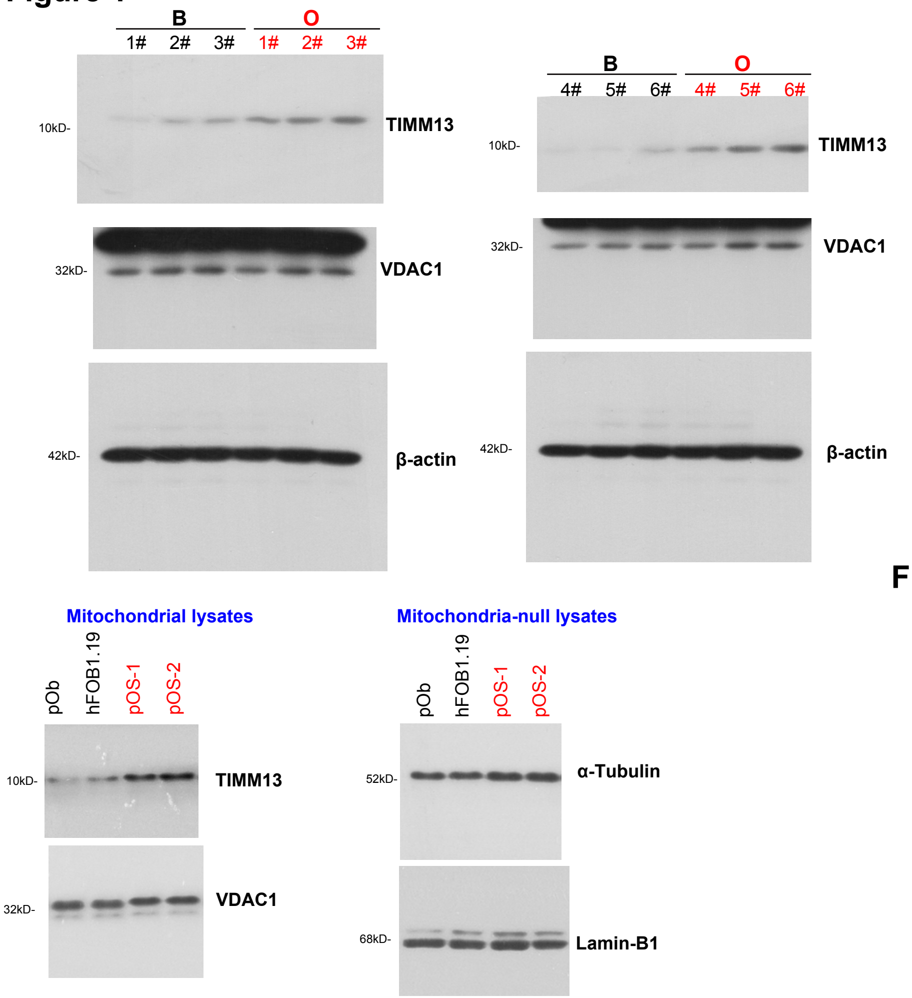


## Figure 5

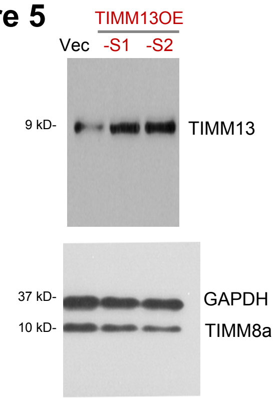


## Figure 2

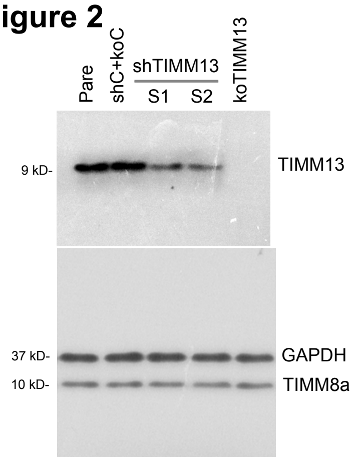


## Figure 6

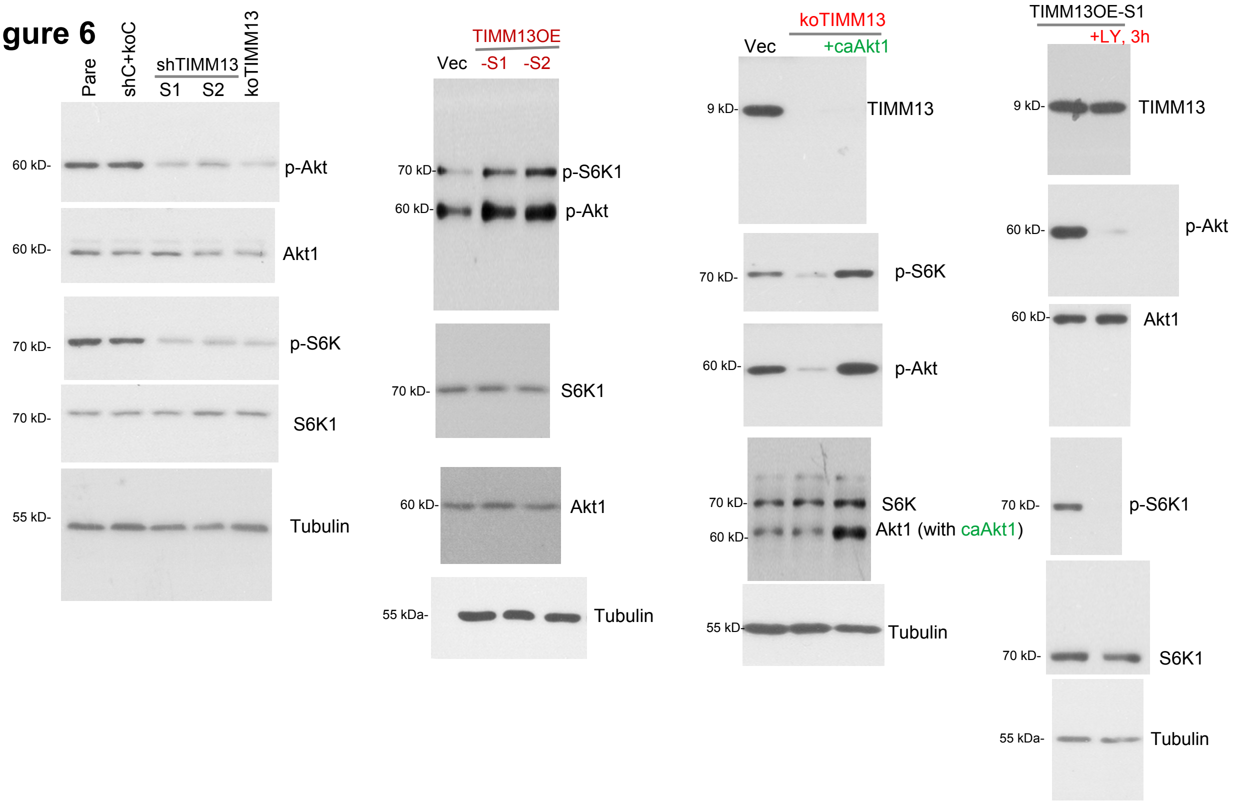


## Figure S2

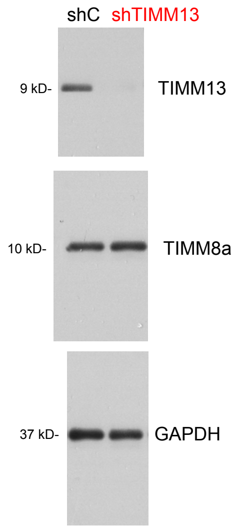

## Figure 7

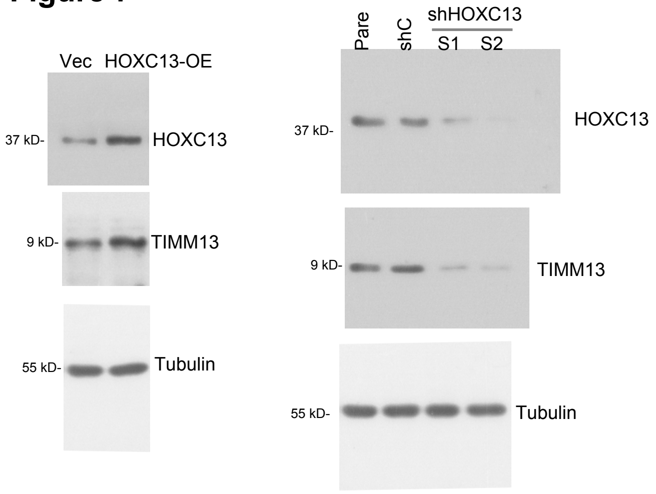


## Figure 8

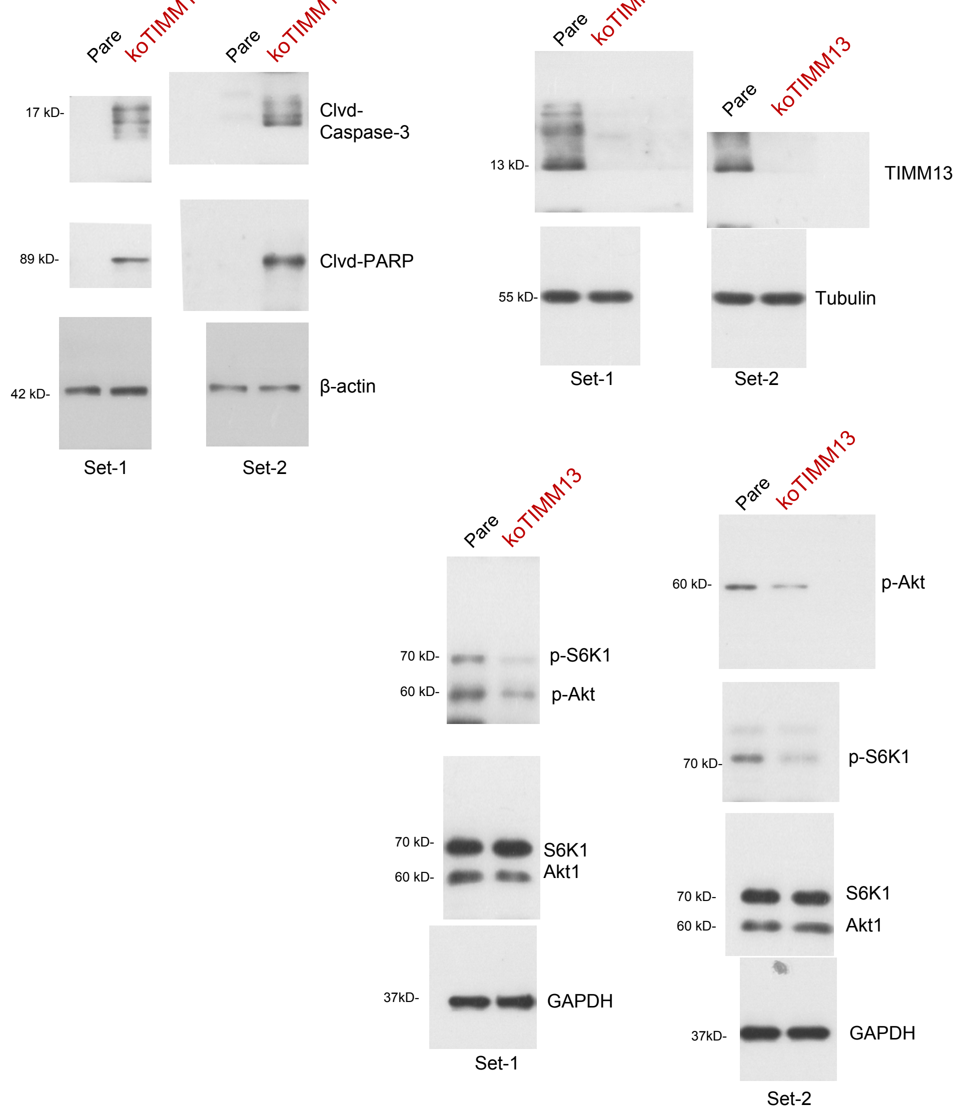

Supplement: Supplementary file 1 — SUPPLEMENTAL Figure 1 [file 41419_2023_5910_MOESM1_ESM.pdf]
