# Supplementary material for: HOXC13-driven TIMM13 overexpression promotes osteosarcoma cell growth
Source: Cell Death Dis. 2023 Jul 5;14(7):398. doi: 10.1038/s41419-023-05910-0 (PMC10322838; doi:10.1038/s41419-023-05910-0)
Supplement: Supplementary file 2 — SUPPLEMENTAL Figure 2 [file 41419_2023_5910_MOESM2_ESM.pdf]

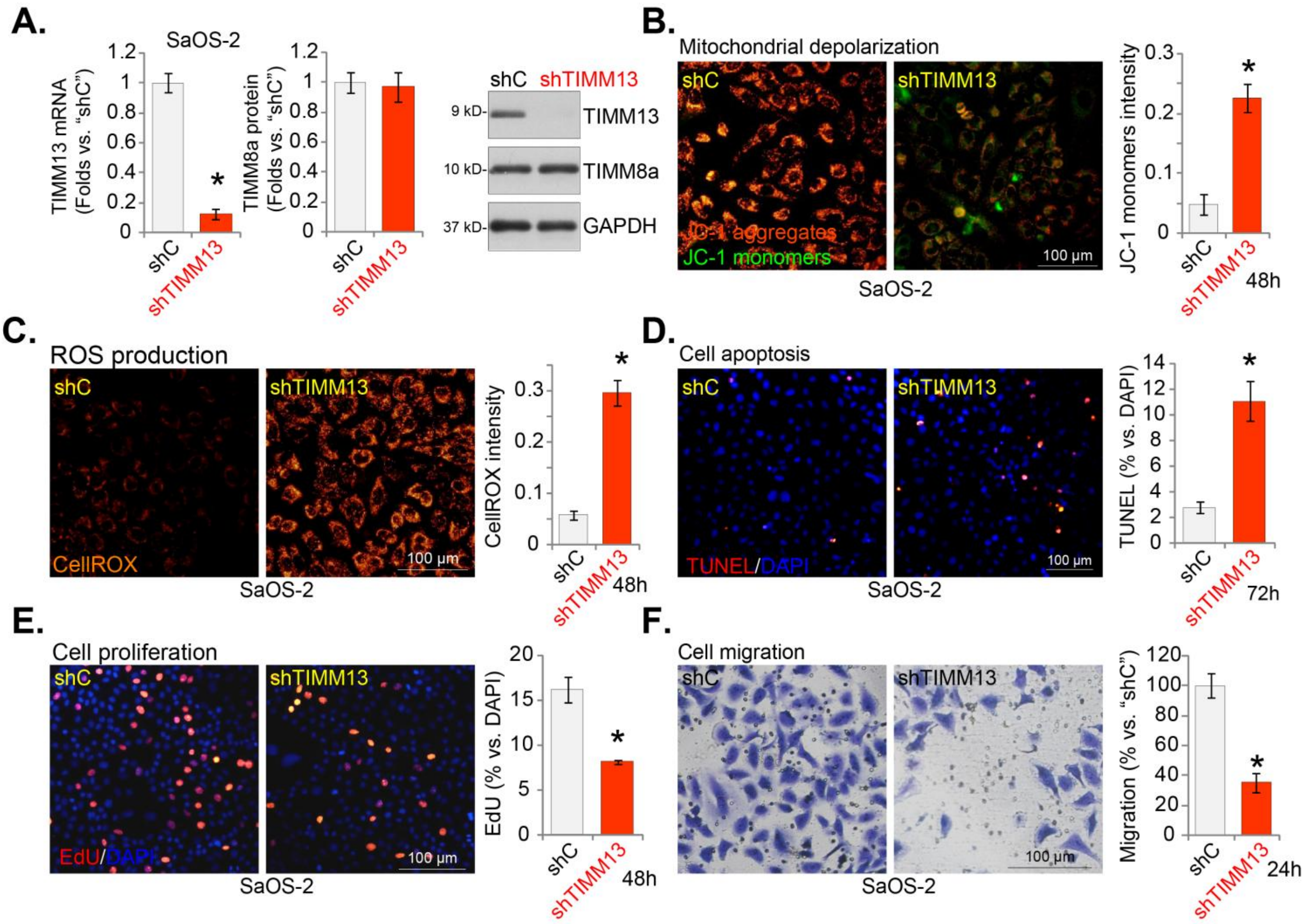

Figure S2. TIMM13 silencing exerts anti-cancerous activity in SaOS-2 cells. SaOS-2 cells were infected with lentiviral shTIMM13-S1 ("shTIMM13") or the lentiviral scramble control shRNA ("shC"), and stable cells were formed following puromycin selection. TIMM13-TIMM8a expression was tested (**A**). Cells were further cultivated under the complete medium for the designated hours, mitochondrial depolarization, ROS production, cell apoptosis, cell proliferation and migration were tested by JC-1 staining (**B**), CellROX staining (**C**), nuclear TUNEL staining (**D**), nuclear EdU staining (**E**) and "Transwell" (**F**) assays, respectively. Error bars stand for mean ± standard deviation (SD, n=5). * ***P*** < 0.05 versus "shC" cells. Experiments in this figure were repeated five times. Scale bar = 100 μm.
